# More than movement: a conceptual exploration of the convergence of social-emotional learning and physical literacy

**DOI:** 10.3389/fspor.2026.1927655

**Published:** 2026-09-30

**Authors:** Kyle Pushkarenko, Liz Durden-Myers

**Affiliations:** 1 School of Human Kinetics and Recreation, Memorial University of Newfoundland, St John's, NL, Canada; 2 School of Education, Bath Spa University, Bath, United Kingdom; 3 School of Health, Education and Science, University of Gloucestershire, Cheltenham, United Kingdom

**Keywords:** culturally sustaining pedagogy, ecological systems, equity, flourishing, holistic development, meaningful physical education, meaningfulness

## Abstract

This paper develops an integrative framework for bringing social-emotional learning (SEL) and physical literacy (PL) into closer conceptual and pedagogical alignment. Rather than treating SEL and PL as separate domains, the paper positions them as mutually reinforcing lenses for understanding how movement experiences can support holistic development across health and physical education, community sport and recreation, outdoor and play-based settings, and lifespan physical activity contexts. Ecological systems theory provides the primary architecture, while socio-constructivist, self-determination, ecological dynamics, and meaningful physical education perspectives explain how embodied learning, motivation, agency, belonging, and adaptive movement competence are cultivated through meaningful activity. Within this integrated view, movement is not limited to fitness, performance, or skill acquisition; it is a relational and culturally situated site through which learners develop empathy, resilience, ethical awareness, confidence, competence, motivation, and understanding. International policy frameworks, including UNESCO's Quality Physical Education guidelines, the OECD's Learning Compass 2,030, and the WHO's Global Action Plan on Physical Activity, further underscore the need for approaches that respond to persistent physical inactivity, widening inequities, and growing concerns about young people's mental health and wellbeing. The framework translates these priorities into design principles that emphasize autonomy, competence, relatedness, equity, cultural relevance, and meaningful engagement. In doing so, the paper proposes SEL–PL integration as an evaluative and conceptual direction for health and physical education and related movement contexts, suggesting that meaningful movement may contribute to identity formation, flourishing, lifelong participation, and more just conditions for wellbeing.

## Introduction

Bringing social-emotional learning (SEL) and physical literacy (PL) together reflects a broader shift in how education, public health, and movement-based practice define success. Rather than focusing primarily on performance indicators, activity metrics, skill outcomes, or test results, there is increasing recognition that schools and community programs must also support young people's capacity to build relationships, navigate social complexity, regulate emotions, develop confidence, and participate meaningfully in active life (1, 2). This shift is especially important for health and physical education (HPE), where learning is not only cognitive or behavioural, but embodied, relational, affective, and culturally situated. Movement experiences can therefore become important contexts through which learners encounter challenge, cooperation, belonging, agency, and ethical responsibility. Bringing SEL and PL into closer conversation helps clarify how these experiences can support not only physical activity participation, but also identity formation, wellbeing, and more equitable conditions for lifelong engagement in movement.

The purpose of this conceptual paper is to clarify how SEL and PL can be integrated as a coherent pedagogical and systems-oriented framework for HPE, while also informing community sport, recreation, outdoor education, active play, health promotion, and other movement contexts. The paper advances HPE scholarship by moving beyond the claim that SEL and PL are compatible to specify why they converge theoretically, what developmental processes they share, and how educators and organizations can design movement experiences that are physically educative, socially and emotionally formative, meaningful, and inclusive.

This clarification is important because PL is sometimes reduced in practice to movement competence, assessment metrics, or sport participation (3–5), while SEL is often operationalized through classroom-based psychosocial programs in ways that can underplay the embodied, relational, and movement-based contexts through which social and emotional capacities are also learned (6–8). The argument developed here is that neither reduction is adequate: PL and SEL are both relational, contextual, and developmental, and HPE offers a distinctive site where they can be enacted together through movement.

Yet, despite widespread recognition of its importance, levels of physical activity worldwide remain worryingly low (9, 10). This is especially concerning, given that research consistently shows that physical activity supports cognitive development, emotional regulation, and overall mental health (11, 12). Noting this, SEL and PL offer complementary ways to understand why these participation gaps continue and how they can be addressed. SEL highlights interpersonal and intrapersonal skills that help people engage, persist, and relate to others (2, 6), while PL focuses on confidence, competence, motivation, and understanding necessary for meaningful participation in movement across life stages (13, 14). Rather than framing inactivity primarily as an individual problem of knowledge, fitness, or willpower, an integrated SEL–PL perspective directs attention to the quality of movement experiences, the social meanings attached to bodies and abilities, and the opportunities, relationships, and environments that either invite or inhibit lifelong participation. When these two perspectives are intentionally combined, they open up possibilities for more equitable, engaging, and responsive learning designs that support each individual's holistic development.

SEL is generally understood as the process through which people learn to understand themselves, manage emotions, set and pursue goals, empathize with others, and build healthy, supportive relationships (15, 16). Transformative SEL further positions identity, agency, and equity as central rather than peripheral to social and emotional development (17, 18). This perspective reminds us that SEL does not happen in a vacuum; it unfolds within cultural, social, and political contexts that influence how young people experience school, community, and movement more broadly (16). Recent HPE scholarship shows that SEL is not simply ‘added’ to physical education lessons. Rather, it emerges through pedagogical models and instructional structures that organize cooperation, responsibility, reflection, and social interaction (7); through student voice, democratic participation, reflective dialogue, and meaning-making in movement (8, 19); and through equity-oriented teacher education and pedagogical practice that attends to identity, relationships, belonging, and culturally responsive facilitation (20). Jones et al.'s (21) SEL model is useful here because it locates skills and competencies within classroom climate, relationships, home and community contexts, implementation supports, and opportunities for skill use, which aligns directly with the ecological argument developed in this paper.

PL is often described as a capacity that develops through continuous interaction with the environment; a way of becoming increasingly confident, capable, and expressive through movement (22). Whitehead (13, 14) describes PL as a holistic capacity rooted in four interconnected domains: affective (motivation and confidence), physical (movement competence), cognitive (knowledge and understanding), and behavioural (regular engagement in activity). These dimensions shape habits, skills, and attitudes that enable individuals to move purposefully and enjoyably across different spaces and stages of life (23). Contemporary PL scholarship further strengthens this holistic interpretation by positioning PL as an evidence-informed determinant of physical activity and health (24), as a developmental framework connecting physical, psychological, social, and creative domains (25), and as a person-centred, lifelong journey shaped through embodied experience, meaning-making, social relationships, and movement contexts (26, 27).

## Conceptualisation and critical debates

The integration proposed here does not resolve the contested status of PL. The literature extensively documents definitional proliferation, competing philosophical commitments, tensions between holistic and component-based accounts, and the risk that policy and assessment practices reterritorialize PL as measurable motor performance (3–5, 23). These debates extend beyond questions of terminology because definitions of PL shape whose movement experiences are valued, which outcomes are assessed, and whether affective, relational, cultural, and embodied dimensions remain visible. As such, this paper explicitly adopts Whitehead's holistic and embodied orientation (14, 22, 28) and positions ecological dynamics as a compatible lens for practice design (29, 30) rather than as an alternative definition of PL. And although SEL does not resolve PL's ambiguity, its conceptual value can make relational, emotional, ethical, and equity-related processes increasingly explicit, and provide criteria against which reductionist appropriations can be questioned.

More specifically, the framework responds to these critiques by refusing to treat PL as a universal checklist of decontextualized competencies or as an outcome that can be inferred from motor performance alone. Its ecological and equity-oriented commitments instead require attention to how institutional norms, assessment practices, cultural meanings, material access, and relations of power shape whose movement is recognized as competent and worthwhile (3–5, 23). At the same time, adding SEL does not automatically protect PL from reductionism. If social-emotional capacities are individualized, standardized, or assessed apart from learners’ identities and conditions of participation, the integrated framework could reproduce the same deficit-oriented tendencies it seeks to challenge. Transformative SEL therefore functions here as a critical safeguard. In other words, agency, belonging, and emotional regulation must be interpreted relationally and with attention to culture, power, and educational equity rather than as neutral attributes located solely within the learner (17, 20). The framework should consequently be judged not only by whether learners acquire named capacities, but also by whether pedagogies and systems expand meaningful participation and redistribute opportunities to influence movement experiences.

## Parallels between SEL and PL

SEL and PL converge across several developmental emphases, particularly when viewed through the pedagogical lens of meaningful physical education. Rather than treating PL as a set of physical capacities and SEL as a separate set of psychosocial competencies, an integrated perspective shows how motivation, confidence, competence, understanding, engagement, relationship skills, and responsible decision-making can develop together through well-designed movement experiences. Table 1 summarizes these points of convergence and clarifies how MPE functions as a pedagogical bridge between PL, SEL, and integrated design practice.

**Table 1:** Conceptual parallels among physical literacy, social-emotional learning, and meaningful PE.

Physical literacy emphasis	SEL emphasis	Meaningful PE bridge	Example pedagogical practice	Ecological mechanism	Anticipated developmental possibility
Motivation and confidence	Self-awareness, social awareness, and willingness to persist	Learners find joy, challenge, and personal relevance in movement	Offer choices, scaffold success, and invite reflection on what movement means.	Need-supportive climates align task challenge with identity, prior experience, and available support.	Greater willingness to explore and re-engage; not assumed to produce sustained participation.
Physical competence	Self-management, emotional regulation, and perseverance	Learners experience competence through appropriately challenging tasks	Differentiate games, dance, outdoor pursuits, and body-management tasks.	Adapted learner, task, and environmental constraints enable exploration and functional solutions.	Adaptive movement competence alongside strategies for managing challenge and frustration.
Knowledge and understanding	Responsible decision-making and ethical reasoning	Learners interpret movement situations and make thoughtful choices	Use prompts about safety, inclusion, tactics, environmental demands, and effects on others.	Dialogue, modelling, feedback, and reflection connect embodied action with social meaning.	More context-sensitive decisions and ethical awareness within movement settings.
Engagement across life contexts	Relationship skills, belonging, and agency	Learners experience social connection and identity affirmation	Connect school, family, community recreation, sport, play, and active-living opportunities.	Coherent relationships and opportunities across ecological settings can reinforce positive movement identities.	Meaningful participation and possible transfer across contexts, contingent on access and cultural relevance.

Meaningful physical education offers a practical link between SEL and PL because student choice, shared norms, real-world task design, and reflective dialogue allow skill development, ethical reflection, teamwork, and agency to occur together. In such environments, learners can feel both competent and connected in ways that are personally meaningful (31). The key point is not that one activity form is inherently meaningful, but that movement experiences become meaningful when they are designed to support competence, connection, choice, challenge, and relevance.

## Conceptual boundaries

Five related constructs play different roles in the framework. Meaningfulness refers to the personally and culturally significant quality of an experience: why participation matters to a learner. Belonging is the relational experience of being recognized, accepted, and able to contribute within a movement setting. Agency concerns the situated capacity to make consequential choices and influence one's participation, rather than choice offered without power. Wellbeing is the broader, multidimensional condition of functioning and feeling well. Flourishing is a more explicitly eudaimonic ideal involving purpose, growth, valued relationships, and the opportunity to live and move in ways one has reason to value (32, 33). Thus, meaningfulness, belonging, and agency are proposed experiential processes; wellbeing and flourishing are possible, distal developmental outcomes. They should not be treated as interchangeable or guaranteed.

## Theoretical orientation: ecological, embodied, and meaningful development

The theoretical argument of this paper is organized primarily through ecological systems theory (34, 35). This theory expands understanding of development by reminding us that learning occurs within nested environments such as classrooms, schools, families, peer groups, communities, and cultural systems, each shaped by norms, relationships, resources, and expectations. For SEL–PL integration, this ecological architecture is especially important because movement experiences are never only individual events; they are shaped by dynamic relations among learners, tasks, environments, social interactions, and the cultural contexts in which movement occurs (29, 30). Within the microsystem, lesson climates, peer relationships, teacher expectations, and material conditions influence whether movement feels safe, meaningful, inclusive, or alienating. Across the mesosystem, connections among schools, families, community sport, recreation, and health-promotion settings can either reinforce or disrupt positive movement identities. At the exosystem level, staffing, policy, assessment, facilities, funding, and professional learning shape what educators and organizations can realistically provide. These conditions are situated within macrosystemic narratives about ability, gender, race, body size, competition, risk, health, and “proper” movement, which influence whose bodies and ways of moving are recognized as legitimate. Across the chronosystem, experiences of inclusion, exclusion, joy, embarrassment, injury, success, and failure accumulate over time, shaping how learners come to understand themselves in relation to movement (34, 35). Framed this way, SEL–PL integration requires attention not only to individual motivation, confidence, competence, or self-regulation, but also to the relational, material, cultural, and temporal conditions that make meaningful movement participation possible.

The resulting arrangement is functional and complementary rather than a rigid, subsuming theoretical hierarchy. Ecological systems theory identifies the levels and conditions within which development occurs (34). Social constructivism explains learning through participation, dialogue, modelling, and shared meaning-making (36). Self-Determination Theory specifies how autonomy, competence, and relatedness may energize engagement within those settings (37, 38). Ecological dynamics guides the manipulation of learner, task, environmental, and sociocultural conditions (29, 30, 39). Meaningful physical education provides the pedagogical bridge through which these ideas become coherent learning experiences (31, 40). PL and SEL are the focal developmental constructs; the other perspectives explain context, process, motivation, design, and enactment. Each is retained because it answers a different explanatory question, reducing theoretical layering and avoiding the claim that any one theory is sufficient.

Socio-constructivist theory helps explain the learning processes that occur inside these ecological systems. Vygotsky (36) highlights how complex psychological abilities such as emotion regulation, reasoning, and social understanding develop through meaningful, guided participation in cultural activities. Applied to SEL and PL, this means that learners do not acquire movement competence, agency, empathy, or responsible decision-making in isolation; they develop these capacities through shared practice, modelling, dialogue, feedback, peer support, and progressive participation in movement cultures (7, 8). A simple example can be seen when teachers scaffold learners’ tactical, interpersonal, and emotional responses during a cooperative challenge. By modelling strategies, asking reflective questions, and gradually releasing responsibility, teachers help students internalize both movement skills and social behaviours. The same principle applies beyond games: across creative, outdoor, aquatic, body-management, active transportation, and culturally specific movement practices, learners co-construct what movement means, how competence is recognized, and how participation can be made more inclusive.

Within this paper, Self-Determination Theory (SDT; 37, 38) functions more modestly as a motivational mechanism within the broader ecological argument rather than as the overarching theory of the paper. Specifically, SDT indicates that motivation, engagement, and adaptive functioning are strengthened when learners experience autonomy, competence, and relatedness within need-supportive movement environments (38, 41). These needs help explain why learners are more likely to engage meaningfully when they can make choices, experience success, and feel that they belong in movement settings. In practice, educators can support these needs by offering choices in roles, inviting students to set personal goals, and involving learners in the design of task constraints.

Meaningful physical education (MPE) provides the pedagogical bridge between theory, curriculum, and instruction. Research on meaningful movement experiences shows that experiences marked by joy, social connection, competence, personal relevance, and challenge support both physical skill development and social-emotional growth (8, 40, 42). The “why” of MPE is that young people are more likely to value movement when it connects to their lives, identities, relationships, and aspirations (31). The “what” includes social interaction, challenge, motor competence, fun, and personally relevant learning (40). The “how” involves student voice, democratic decision-making, cooperative learning, reflective questioning, peer feedback, culturally responsive activity choices, and repeated opportunities to deepen experience over time (8, 19, 43).

Building on ecological dynamics accounts of PL as relational, adaptive, and context-sensitive, movement learning can be understood as emerging through dynamic interactions among learners, tasks, environments, and sociocultural conditions (29, 30). Within the broader ecological architecture, ecological dynamics functions as a practice-oriented lens for designing responsive movement experiences. Constraints-led approaches show how educators can adapt task, environmental, and learner constraints to support exploration, problem-solving, and participation (39, 44). Variability, representative tasks, and co-design become not just instructional techniques but opportunities to practise collaboration, empathy, and ethical decision-making while developing movement-related abilities and confidence (45). Task constraints can be adapted across educational gymnastics and balance-oriented sequences (46), circus arts activities (47), outdoor and cooperative adventure tasks (48), dance composition and creative movement (49), and inclusive fitness or adapted activity circuits (50). In these contexts, learners are invited to explore, negotiate, communicate, and solve movement problems in ways aligned with both PL and SEL.

## Contribution to knowledge

The novelty within the PL–SEL integration lies neither in claiming that movement simply has social-emotional benefits nor in combining two lists of competencies. Rather, it affords four specific contributions. First, it locates SEL–PL convergence within a nested ecological architecture, shifting explanation from individual attributes to relations among learners, tasks, relationships, institutions, cultures, and time (34, 35). Second, it differentiates the functions of the supporting theories: social constructivism explains how capacities are co-constructed (36); Self-Determination Theory specifies a motivational pathway (37, 38); ecological dynamics informs task and environmental design (29, 30); and meaningful physical education translates these mechanisms into pedagogy (31, 40). Third, it identifies meaningfulness, belonging, agency, and psychological-need satisfaction as mediating processes through which movement experiences may connect SEL capacities with PL development (38, 40, 41). Fourth, it frames equity and cultural relevance as constitutive design conditions rather than desirable downstream outcomes (17, 18, 20). This architecture advances beyond parallel or additive accounts by specifying where integration occurs, how it may operate, and which propositions require empirical examination. This contribution is consistent with theory-synthesis and model-oriented conceptual scholarship, in which originality may arise through relating previously separate literatures, explicating their connections, and producing a framework that reorganizes understanding and directs subsequent inquiry (51, 52).

The model proposes a reciprocal sequence rather than a linear causal chain. Belonging may support PL when recognition, trust, and legitimate participation reduce social threat and expand opportunities to explore movement; successful participation may, in turn, deepen belonging. Agency may support sustained engagement when learners can shape goals, roles, task constraints, and interpretations of success, thereby strengthening autonomy and personal relevance. Social-emotional capacities interact with movement competence during action: emotion regulation can help learners remain engaged under challenge, empathy and communication can coordinate shared movement problems, and embodied success or difficulty supplies experiences through which self-awareness is revised. Meaningfulness is proposed to mediate these relations by connecting what occurs in an activity to identity, values, relationships, and future possibilities. These are theory-informed conceptual propositions not empirically established mediators or invariant causal pathways. Their existence, direction, strength, sequence, reciprocity, and transfer across settings require empirical examination.

Taken together, the proposed mechanisms position SEL–PL integration as the design of lived movement experiences rather than the alignment of competency lists. Experiences characterized by appropriate challenge, social support, cultural relevance, learner influence, and opportunities for reflection may connect movement participation with identity, belonging, agency, and a salutogenic sense of coherence (8, 22, 33, 53). Their developmental significance is expected to depend on whether learners can understand what is being asked, access sufficient support, and find personally or culturally meaningful reasons to participate. Accordingly, integrated design may include respectfully community-led Indigenous or local movement practices, community-connected dance, place-based outdoor activity, circus or balance tasks, and cooperative challenges that value communication and trust alongside movement competence (54, 55). These examples illustrate possible design conditions rather than universally effective activity forms; whether and how they support wellbeing or flourishing remains an empirical question (32).

## Complimentary constructs

The conceptual overlap between SEL and PL becomes particularly evident when examining outcomes. Decades of research on school-based SEL initiatives consistently demonstrate improvements in social behaviour, fewer conduct issues, and gains in academic achievement (6). Likewise, studies in physical activity and youth sport highlight how well-designed programs can boost mental health, executive functioning, and broader positive youth development (11, 56, 57). HPE-specific scholarship adds further nuance by showing how SEL can be embedded in movement-based pedagogies, including cooperative learning and other model-based instructional structures (7), student voice, democratic decision-making, reflective dialogue, and meaningful physical education (8, 19), restorative approaches to relationships, conflict, and harm (58), and teacher education that explicitly attends to equity and social justice (20). When these approaches are deliberately combined, movement experiences that include inclusive norms, reflective moments, cooperative challenges, and opportunities for student voice can support social-emotional growth and physical skill development simultaneously.

Several practical examples illustrate how SEL and PL work together across diverse movement contexts. Creative movement can foster emotional expression and social connection while also developing rhythm, coordination, and confidence; evidence from dance and movement therapy supports these outcomes (59). Outdoor pursuits and adventure-based activities can invite learners to manage risk, cooperate, communicate, and develop confidence in unfamiliar environments (48). Circus arts and body-management tasks can further broaden understandings of balance, coordination, creativity, trust, progressive challenge, and physical literacy beyond conventional sport-based forms (47). Inclusion-focused programs, such as Unified Sports, further reinforce this point. When thoughtfully coached, mixed-ability teams tend to foster a sense of belonging, enhance attitudes towards inclusion, and promote fitness gains for all participants (60, 61). Across these examples, progressive challenge, supportive feedback, adapted constraints, and representative tasks can develop PL while also nurturing social-emotional growth in caring, culturally responsive environments.

Collectively, these findings suggest a set of design principles for integrated SEL–PL learning. Activities should mimic real-world movement scenarios but remain adaptable to accommodate different skill levels and learning needs. Ecological dynamics offers valuable guidance in this regard. By modifying rules, space, time, equipment, sensory demands, levels of risk, or participant roles, educators can promote exploration and adaptive coordination. For example, a teacher might design a balance-and-travel sequence with varied equipment, learner-selected challenge levels, peer feedback on safety and encouragement, and a reflective prompt about frustration, confidence, and support. Small-sided games with rotating leadership roles can also support tactical thinking, communication, and emotional regulation, but they should be treated as one option within a wider movement repertoire. Incorporating structured reflection or peer feedback also helps make social learning visible, reinforcing the SEL components embedded within the activity.

Another crucial design principle is to support learners’ basic psychological needs for autonomy, competence, and relatedness (37, 38). Autonomy can be supported through meaningful choice; competence through transparent, developmentally appropriate, and optimally challenging goals; and relatedness through inclusive interaction, peer mentoring, and shared objectives. When these conditions are present, learners are more likely to remain engaged in movement and practise SEL capacities such as empathy, agency, and responsible decision-making (11).

A third fundamental principle is equity. Programs that affirm students’ identities and lived experiences draw on a wider range of movement practices (e.g., street games, cultural dances, outdoor skills) and, in doing so, broaden what is recognized as competence (16). Celebrating holistic development across the physical, affective, cognitive, and social domains helps to ensure that all learners can make progress. Paying attention to participation, leadership, and rule-setting helps ensure that movement spaces promote agency and belonging rather than inadvertently foster exclusion. Culturally sustaining and identity-affirming practices can support engagement when paired with relational trust, meaningful choice, appropriate challenge, and opportunities for learner voice (8, 16, 17, 19, 20). When learners see their cultures and bodies reflected in activities, movement is less likely to feel alienating and more likely to connect with belonging, recognition, motivation, and personal relevance.

Meaningful integration also extends beyond the classroom. Because PL develops through accumulated movement experiences across the life course, schools, after-school programs, recreation groups, sport organizations, families, and health-promotion settings can either reinforce or fragment SEL–PL development (13, 14). When these settings share common language and goals, they can create a more coherent ecology of experiences that supports empathy, agency, identity development, wellbeing, and lifelong movement participation (57). From this wider perspective, SEL and PL can be treated as developmentally interdependent rather than assumed to be inseparable. By connecting meaningful movement experiences to personal identity and contribution, integrated SEL–PL design may provide a foundation for holistic development and human flourishing.

## International and cultural applicability

The framework is informed predominantly by scholarship produced in Western, English-speaking settings and should not be universalized without local interpretation. SEL frameworks themselves can privilege Global North assumptions about the individual, emotion, communication, and desirable social conduct, while movement, competence, wellbeing, interdependence, and flourishing may be understood differently across cultures (62–64). In Global South, Indigenous, rural, displaced, and low-resource contexts, integration should therefore begin with community-defined purposes, locally meaningful movement practices, available materials and spaces, and culturally legitimate ways of expressing emotion, relationship, responsibility, and agency. Ecological responsiveness requires co-design with learners, families, knowledge holders, and community organizations; translation of language and constructs; and scrutiny of whether imported SEL or PL frameworks reproduce deficit assumptions. The model offers organizing questions, not a context-free program.

## Implications for policy, systems, and practice

Integrating SEL and PL has important implications for policy, system design, and daily practice. UNESCO, OECD, and WHO frameworks collectively emphasize inclusive curricula, student agency, life skills, supportive environments, and whole-system approaches to wellbeing (12, 65–68). These frameworks suggest that embedding PL outcomes within SEL practices, enhancing educator preparation, co-developing curricula, and integrating measures of inclusion and agency into accountability systems are essential steps. They collectively position embodied, relational learning as central to meaningful education across diverse settings.

Achieving this integrated vision also requires rethinking how SEL and PL are assessed. Traditional methods often treat them separately, with SEL evaluations tending to focus on social and emotional competencies, psychosocial outcomes, or program effects (69, 70), and some applications of PL assessment being interpreted narrowly through motor competence, fitness, or fundamental movement-skill scores rather than the construct's fuller physical, affective, cognitive, behavioural, and social dimensions (4, 71, 72). A more holistic approach employs multiple methods and perspectives, grounded in theory, to give educators practical, actionable insights. Assessments should be developmentally appropriate and provide meaningful guidance for teaching rather than primarily serving accountability purposes (13). Strategies such as co-developing success criteria, student voice, peer feedback, reflective journals, and individual goal setting can help teachers and students interpret progress formatively. Learners might document how they approached a challenge, what emotions emerged, how peers supported one another, and what adaptations helped them experience competence, while teachers triangulate these reflections with observation, peer dialogue, and movement performance. Assessment tools and criteria must also be culturally responsive, attentive to diverse learners and contexts, and used to broaden access and enhance motivation rather than restrict participation (16, 69, 70).

A feasible integrated assessment sequence could combine: (a) learner-selected movement goals; (b) brief experience-sampling prompts about meaning, belonging, confidence, emotional response, and perceived choice; (c) teacher observation of adaptive movement solutions, cooperation, help-seeking, persistence, and ethical decision-making; (d) peer dialogue focused on contribution and inclusion; and (e) portfolios containing reflections, task adaptations, and evidence of learning across contexts. Existing PL and SEL instruments may inform this process, but systematic reviews caution that no single measure adequately represents either multidimensional construct and that purpose, feasibility, validity, age, culture, and consequences of use must guide selection (4, 69–72). Indicators of meaningful participation should therefore include whether learners had genuine choice, could contribute in valued ways, experienced recognition and appropriate challenge, and could explain the significance of the experience. These sources should be triangulated formatively rather than collapsed into a decontextualized SEL–PL score.

Integrating SEL and PL also involves confronting longstanding inequities that influence physical activity and sport participation. Barriers related to gender norms, ability biases, heteronormativity, racialized tracking, poverty, language, geography, and body-size stigma have historically limited involvement for many young people. Transformative SEL offers a way forward by viewing competence as culturally embedded and co-constructed, highlighting learners’ unique identities, languages, and community movement traditions as strengths (16, 17). Practically, this means creating environments where psychological and physical safety are shared responsibilities, where curricula intentionally include culturally relevant games, dances, outdoor activities, and non-sport movement forms, and where competence is recognized in multiple forms, not solely through traditional measures. Concrete action steps include auditing whose movement practices are represented; co-designing tasks, norms, and success criteria with students; rotating leadership roles so leadership is not limited to athletic dominance; using reflection to examine belonging and agency; structuring peer feedback around encouragement and specificity; and adapting equipment, space, grouping, pace, and assessment criteria so learners with different bodies, abilities, histories, and cultural experiences can contribute meaningfully.

Programs like Unified Sports demonstrate how inclusive norms and reflection can support belonging, attitudes toward inclusion, and fitness outcomes when intentionally coached (60, 61). However, SEL–PL integration should not be imagined only through sport teams. Non-competitive, expressive, outdoor, adventure-based, rhythmic, and self-paced movement opportunities are also needed so learners can experience competence, connection, and identity affirmation in multiple ways.

Sustaining SEL–PL integration also relies on preparing educators to operate in complex, dynamic environments. Teacher education should prepare educators to adapt tasks, interpret social and physical cues, provide meaningful feedback, and build movement environments where learners experience safety, belonging, and agency. Pre-service programs can support this by blending coursework in motor learning, pedagogy, and human development with practical experiences that foreground autonomy-supportive and relational practice. For practising educators, professional learning communities can provide opportunities for video analysis, reflective dialogue, and refinement of assessment practices that support both physical and social-emotional development. Recent PETE research supports this direction by emphasizing that teacher educators need to prepare future physical educators to understand SEL as equity-oriented, pedagogically enacted, and inseparable from curriculum design, power-sharing, relationships, and reflective facilitation (20, 73). When these capacities are systematically developed, educators are better prepared to align their practices with UNESCO, OECD, and WHO priorities in scalable and sustainable ways.

## Conceptual limitations

The framework is most plausible where educators can modify tasks and environments, share some decision-making power, support relationships, and value development beyond performance. It may function differently, or even fail, within highly competitive climates, tightly standardized curricula, performance-dominated assessment systems, unsafe or exclusionary peer cultures, and settings with limited time, facilities, equipment, staffing, or professional learning. Choice can be superficial when acceptable outcomes remain predetermined; cooperation can reproduce status hierarchies; reflection can expose learners when psychological safety is absent; and culturally themed activities can become tokenistic when separated from community knowledge. Competitive activity is not inherently incompatible with the framework, but selection, ranking, and public comparison may undermine relatedness, agency, and meaningful participation unless intentionally moderated. Nor should SEL–PL integration be expected to compensate for structural barriers such as poverty, ableism, racism, gender regulation, inaccessible facilities, or exclusionary policy. Finally, the framework is a conceptual synthesis rather than an empirically validated causal model. Its constructs overlap, proposed mechanisms may be bidirectional, and implementation will be conditioned by culture, developmental stage, setting, and resource ecology.

Future research should examine how SEL–PL integration operates across settings, populations, and systems. Design-based studies are needed to refine integrated learning units across school, community, recreation, and sport contexts. Mixed-methods research can examine relationships among movement competence, SEL development, mental health, and meaningful engagement, while qualitative and participatory research can illuminate how learners experience belonging, agency, meaning, confidence, and exclusion. System-level research is also needed to understand how policy alignment, professional learning, and community partnerships support long-term implementation. Collectively, this work can provide policymakers, practitioners, and researchers with evidence for creating equitable, system-wide approaches that promote competence, joy, agency, and social-emotional growth throughout life.

The framework generates five priority questions. (a) Under what conditions do meaningfulness, belonging, agency, and psychological-need satisfaction mediate relations between integrated movement experiences and PL/SEL development? (b) How do these mechanisms differ across age, disability, gender, culture, socioeconomic position, and movement setting? (c) Which combinations of task adaptation, learner voice, reflection, and relational practice are feasible and effective in resource-constrained contexts? (d) How do competitive, assessment-intensive, and culturally distinct environments alter or interrupt the proposed processes? (e) Can longitudinal, participatory, and mixed-method designs identify reciprocal change among movement competence, social-emotional capacities, identity, and sustained participation without reducing the constructs to a single score? These questions position Figure 1 as a revisable heuristic comprising theory-informed propositions and specify an empirical program rather than a validated model or confirmed theory of change.

**Figure 1:**
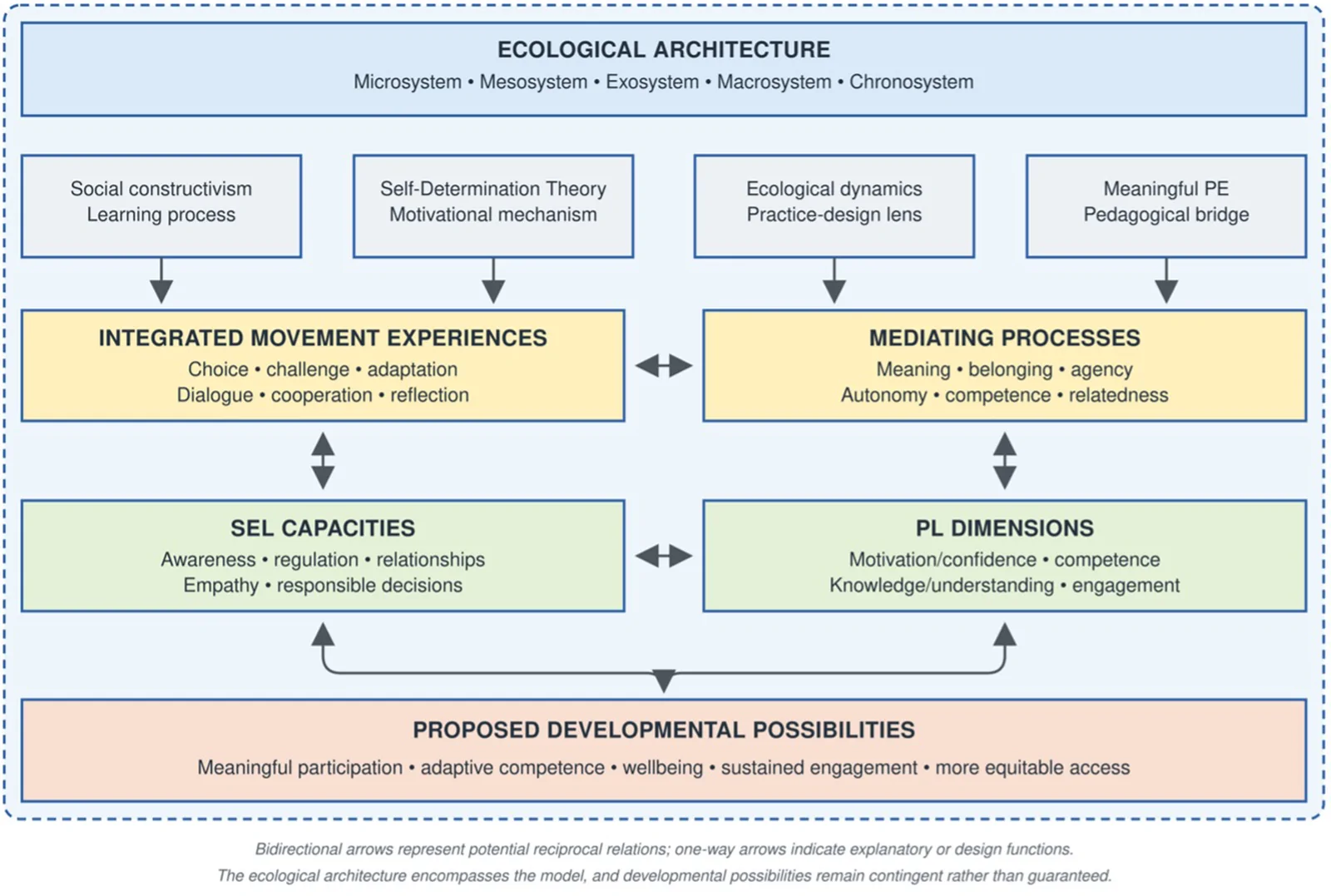
Heuristic conceptual model of SEL–PL integration. Ecological conditions are proposed to shape movement experiences; differentiated theoretical lenses specify possible explanations for learning, motivation, design, and pedagogy; and meaningfulness, belonging, agency, and psychological-need satisfaction are hypothesized mediating processes linking SEL and PL. Arrows represent proposed, potentially reciprocal relations rather than established causal effects. All relations, mechanisms, and developmental possibilities require empirical examination.

## Conclusion

At their core, SEL and PL both aim to support the growth of young people who are capable, caring, and able to contribute meaningfully to their communities. Their intersection highlights the central claim of this paper: movement is a powerful context for integrated physical, social, emotional, cognitive, cultural, and ethical development. Against the backdrop of inequities, physical inactivity, and concerns about young people's wellbeing, SEL–PL integration offers an evaluative conceptual direction for HPE and related movement contexts. When educators design movement experiences that promote autonomy, competence, relatedness, equity, and meaningful engagement, they create environments where students can experience joy, connection, agency, and purpose. The contribution of the framework proposed here is to locate this integration within an ecological account of development, where learners’ relationships with movement are shaped across classrooms, schools, families, communities, policies, cultures, and time. Framed this way, movement becomes an essential part of human life rather than merely a pursuit of fitness, performance, or isolated skill development.
